# Use and Effects of Eltrombopag in Pregnant Woman With Difficult-to-Treat Idiopathic Thrombocytopenic Purpura (ITP)

**DOI:** 10.1155/crh/2509169

**Published:** 2025-07-24

**Authors:** Graciella Calsolari Figueiredo de Farias, Guilherme Tavares de Sá, Renata Vergueiro Mercadante, Bianca Stefanello, Rossana Pulcineli Vieira Francisco, Ana Maria Kondo Igai

**Affiliations:** ^1^Department of Obstetrics and Gynecology, Division of Obstetrics, Faculty of Medicine, University of São Paulo, São Paulo, Brazil; ^2^Department of Obstetrics and Gynecology, Division of Obstetric Clinic, Faculty of Medicine, University of São Paulo, São Paulo, Brazil

**Keywords:** hematologic therapy, maternal outcomes, refractory thrombocytopenia, thrombopoietin receptor agonist

## Abstract

**Objective:** A case report of a pregnant patient with idiopathic thrombocytopenic purpura refractory to clinical treatment, who underwent childbirth in the context of a difficult-to-control disease.

**Method:** Data were collected through medical record survey and literature review.

**Final Considerations:** The case reported regards a complex clinical condition involving management of maternal–fetal well-being in the context of severe hematological disease, regarding ideal therapy options and best moment for delivery indication, minimizing risk of prematurity and adverse events from underlying pathology.

**Summary**

• Alternative drug option for refractory thrombocytopenic idiopathic purpura treatment in pregnancy.
• Use of eltrombopag for refractory thrombocytopenic idiopathic purpura treatment in pregnancy.
• The need for individualized management of pregnant women with idiopathic thrombocytopenic purpura.

## 1. Introduction

Platelet disorders are the second most common disease found during pregnancy after anemia [1]. The normal platelet count in the general population varies between 150 × 10^3^ and 400 × 10^3^/mm^3^. In pregnant women, thrombocytopenia is defined as a platelet count below 150 × 10^3^/mm^3^ [1]. Thrombocytopenia of around 10% can occur in normal pregnancies, especially in the third trimester [2, 3]. A study conducted by Burrows and Kelton involving approximately 15,000 women and their newborns found that thrombocytopenia (platelet count below 150 × 10^3^/mm^3^) occurred in 6.6% of patients, being most frequently caused by gestational thrombocytopenia (74% of cases), 21% of cases, was associated with hypertensive complications of pregnancy and immunological causes, including immune thrombocytopenia (ITP) in 4% of cases [4]. The following case report regards a pregnant woman with ITP refractory to several lines of treatment and the obstetric outcome.

## 2. Case Report

A 16-year-old pregnant woman, first-time mother, with a gestational age of 30 weeks and 3 days, dating from the date of the last menstrual period (LMP), with no known pathological history, seeks emergency care referred by health clinic where she underwent prenatal care due to easily appearing bruising, associated with thrombocytopenia of 11 × 10^3^/mm^3^ platelets for the last 2 months. According to hospital records, the patient was hospitalized and underwent investigation for infectious and autoimmune causes associated with thrombocytopenia (HIV, syphilis, dengue fever, and autoimmune antibodies), the result of which was negative. Treatment for ITP with prednisone 1 mg/kg/day began on 03/20/2023. However, 3 days after admission, her platelet count dropped to 5 × 10^3^/mm^3^, when she was prescribed 7U of platelet concentrate. On 03/27/2023 and 03/28/2023, she resumed taking prednisone at the same dose (of 60 mg), associated with IVIG 0.4 mg/kg, which did not show a significant increase in platelet count, at the time with a value of 7 × 10^3^/mm^3^. Given the hypothesis of immunoglobulin-refractory thrombocytopenia, pulse therapy with methylprednisolone 1 g/kg/day for 3 days was carried out. Due to the lack of response to pulse therapy, treatment with eltrombopag 25 mg/day was instituted on 04/03/2023, with platelet values lower than 2 × 10^3^/mm^3^. On 04/10/2023, she was transferred to our service, being admitted to a high-risk obstetrics ward with platelet count of 2 × 10^3^/mm^3^. Initially, the patient continued prednisone at 60 mg/day, while eltrombopag was discontinued, and intravenous immunoglobulin was prescribed at a dose of 70 g/day for two days (April 11 and 12, 2023). The therapeutic response was unsatisfactory, with a maximum platelet count of 23 × 10^3^/mm^3^ (April 14, 2023), followed by a rapid drop to 3 × 10^3^/mm^3^ on April 18, 2023. Due to the inadequate response to IVIG, eltrombopag was reintroduced on April 18, 2023, at a dose of 50 mg/day. The prednisone dose was progressively reduced to 40 mg/day (April 19, 2023), 20 mg/day (May 1, 2023), and 10 mg/day (May 6, 2023) until the date of delivery (May 15, 2023).

The patient also received an additional IVIG dose (70 g) on May 3, 2023, and on the day before delivery (May 14, 2023), she was administered another course of IVIG (30 g/day for 5 days). Despite the combined use of IVIG, eltrombopag, and prednisone, the maximum platelet response achieved was 20 × 10^3^/mm^3^ on the day of delivery, with a rapid decline to 3 × 10^3^/mm^3^ on May 16, 2023 (Table 1).

On May 19, 2023, the eltrombopag dose was increased to 75 mg/day. The patient was discharged on May 21, 2023, on prednisone 5 mg/day and eltrombopag 75 mg/day.

## 3. Childbirth

Due to lack of response to clinical treatment, maternal–fetal medicine specialists indicated elective resolution of the pregnancy at term, with gestational age of 36 and 6/7 weeks. The cesarean section was indicated due to the impossibility of performing neuraxial anesthesia for assistance during labor due to thrombocytopenia below 70 × 10^3^/mm^3^. On 05/15/2023, according to prior discussions with hematology team, the patient received an infusion of immunoglobulin, followed by a transfusion of a unit of platelets via apheresis, and underwent the proposed procedure under general anesthesia, without major complications. There was uterine hypotony managed with oxytocin, ergotamine, and misoprostol. At 08:07 on 05/15/2023, a baby boy was born, weighing 2450 g, Apgar 8/9/10. After giving birth, the patient was monitored and showed satisfactory progress. The use of eltrombopag and IVIG was maintained for 5 days total (until 05/19/2023). On the first postpartum, patient had platelets of 3 × 10^3^/mm^3^ and no increased bleeding, good-looking surgical wound, and physiological lochia. Kept under clinical and laboratory evaluation in the postpartum period, after stabilization of platelet levels, the patient was discharged on 05/21/2023 to continue care on an outpatient basis and advised to maintain the use of eltrombopag (Table 2).

## 4. Newborn Evolution

A late preterm male newborn had first platelet count of 32 × 10^3^ mm^3^. On his second day of life, he received 15 mL/kg of platelet concentrate, as well as 0.5 g/kg of IVIG. According to hospital records, there were no alterations on physical examination, except for a hematoma in the cervical region, which remained stable during hospitalization. On the fourth day of life, he had received 2 g/kg of IVIG in multiple doses (on May 16, 17, and 18/2023) presenting a maximum platelet value of 37 × 10^3^/mm^3^; he did not receive further platelet transfusions and underwent phototherapy due to neonatal jaundice. Examinations were performed on 05/18/2023: Fontanelle ultrasound was normal; ECG showed no abnormalities; and echocardiogram with Doppler revealed a patent foramen ovale. At the end of the first week of life, With a birth weight of 2. 498 grams, the newborn was discharged from the hospital for outpatient follow-up.

## 5. Patient Evolution After Discharge

An outpatient return for postpartum follow-up was scheduled at the HC-FMUSP Obstetrics and at the HC-FMUSP Hematology outpatient clinic, but the patient did not return. Telephone contact was made and the appointments rescheduled, but the patient did not attend again; we believe she returned to the reference hospital. She also did not return with the baby for outpatient follow-up at the Children's Institute of the Hospital das Clínicas, Faculty of Medicine, University of São Paulo (ICr-HCFMUSP).

## 6. Discussion

ITP occurs in approximately 2 in every 1000 pregnant women [2]. It is a relatively common autoimmune disease caused by antiplatelet antibodies that can cross the placental barrier and cause fetal thrombocytopenia [2]. The pregnant woman may have a prior history of ITP or the disease may arise during pregnancy [1]. It usually manifests gradually, evolving over years with periods of improvement and exacerbation [5].

The typical clinical presentation includes mild bleeding, such as petechiae and ecchymoses on areas exposed to trauma [6]. Gingival bleeding and epistaxis may also occur but are less frequent. Severe bleeding (involving the central nervous system, digestive, and urinary systems) is rare and tends to occur when platelet counts fall below 10 × 10^3^/mm^3^ [7]. Diagnosis is based on clinical history and exclusion of other more common causes [8]. Laboratory tests may identify IgG-class antiplatelet antibodies linked to platelet glycoproteins (IIb, IIIa, Ib, and IX) [8], though their sensitivity is low, they do not correlate with clinical presentation, and are not recommended for definitive diagnosis [8].

### 6.1. Treatment Decision

Treatment should be conducted in conjunction with specialists in Obstetrics, Hematology, and Neonatology. It is recommended to initiate treatment if the platelet count reaches between 20 × 10^3^ and 30 × 10^3^/mm^3^, even in the absence of clinical manifestations, or at higher values if bleeding is present [3]. Approximately 50% of pregnant women may experience progressive platelet decline during pregnancy, particularly in the third trimester [5]. The initial recommended therapy is corticosteroids (prednisone 1 mg/kg/day), with variable responses [2]. Some patients begin with lower doses, such as 20 mg/day, to minimize adverse effects (worsening or development of gestational diabetes, hypertension, sleep disturbances, and gastritis) [3]. The average time to response is approximately 16 days.

### 6.2. Response to Therapy

IVIG can be used at a dose of 0.4 mg/kg/day for 5 days or 1 g/kg/day for 2 days [3]. A response is usually seen within 24–72 h, with platelet elevation maintained for up to one month [9]. In case of therapeutic failure, splenectomy may be considered in the second trimester [10]. There is no consensus on second-line treatments such as azathioprine and cyclosporine in patients with platelet counts between 20 × 10^3^ and 30 × 10^3^/mm^3^ near delivery, as these therapies have slow responses that may take 3–6 months [2, 9].

### 6.3. Use of Eltrombopag

Recently, the thrombopoietin receptor agonist (rhTPO), eltrombopag, has been prescribed outside of pregnancy, orally, at doses of 50–75 mg/day [11]. It can cross the placental barrier due to its low molecular weight [12], and its side effects include nausea, diarrhea, respiratory and urinary infections, increased risk of deep vein thrombosis, and hepatotoxicity [5]. The effect of eltrombopag during pregnancy is not fully established, as there are no clinical studies evaluating its transplacental passage. Its use has recently been recommended in cases refractory to conventional first-line therapies [5, 13, 14].

### 6.4. Fetal Safety and Monitoring

Published studies have reported no congenital anomalies, even with use during the first trimester (1 case) and prepregnancy use (1 case). Some cases of fetal growth restriction have been reported [12]. Several studies indicate that severe fetal thrombocytopenia leading to hemorrhage and death is uncommon and unpredictable [3]. Thrombocytopenia occurs in 12%–28% of neonates born to mothers with ITP, with no correlation between maternal and neonatal platelet counts [12]. The risk of birth with platelets below 50 × 10^3^/mm^3^ is 10%–15% [2] and rarely results in intracranial hemorrhage [6, 9, 10]. The risk of platelets below 20 × 10^3^/mm^3^ is 1% (retrospective studies) to 5% (prospective studies) [2]. The risk of severe fetal hemorrhage resulting in death is 0%–1.5% [15].

## 7. Conclusion

A greater number of studies are necessary to determine the efficacy and safety of eltrombopag during pregnancy, despite its recent recommendation as a treatment option in refractory cases of ITP. There are few reports in the literature involving pregnant women; of the six reported cases [4], four achieved a positive response with platelet counts exceeding 50 × 10^3^/mm^3^ at delivery, while two did not achieve a satisfactory response. In the presented case, the expected increase in platelet count did not occur.

A recent article published in 2023 reported 45 cases in which eltrombopag and romiplostim were administered during pregnancy for ITP, with thrombocytopenia improvement within one week in 86.7% of cases reported [16]. Similar results were observed by Kong et al. [17], who used rhTPO in pregnant women with refractory ITP, obtaining a positive response in about 80% of cases. These findings suggest that in the third trimester of pregnancy, there may be treatment failure with conventional therapies such as corticosteroids and immunoglobulin, complicating labor management and precluding neuraxial anesthesia [18]. Michel et al. also reported a 77% success rate with the use of eltrombopag and romiplostim in 15 pregnant patients with refractory ITP [19].

In our case, we did not achieve success with eltrombopag, and the other rhTPO (romiplostim) is not available in our center. This case may represent an example of extreme refractoriness to medical treatment. Currently, eltrombopag is not considered first-line treatment for ITP but could be used as an alternative in refractory cases. Further studies are needed for cases that do not respond to first- and second-line treatments in ITP [14, 18, 20–23].

## Figures and Tables

**Table 1:** Thrombocytopenia case data.

Date/GA	Management/event	Platelets (× 10^3^/mm^3^)	Observations
∼28 weeks	Thrombocytopenia identified during prenatal care	11.0	Easy bruising
03/20/2023	Started prednisone 1 mg/kg/day	5.0	Transfused with 7 units of platelets
03/27–28/2023	IVIG 0.4 mg/kg + prednisone 60 mg	7.0	No significant response
After IVIG	Methylprednisolone pulse therapy 1 g/day × 3 days		No response
04/03/2023	Started eltrombopag 25 mg/day	< 2.0	Persistent severe thrombocytopenia
04/10/2023	Transferred to tertiary hospital	2.0	High-risk obstetric admission
04/11/2023		1.0	
04/12/2023		1.5	
04/13/2023		9.0	
04/14/2023		23.0	
04/17/2023		13.0	
04/18/2023		3.0	
04/19/2023		4.4	
04/24/2023		1.0	
04/26/2023		0.6	
04/27/2023		1.4	
04/28/2023		3.4	
05/02/2023		0.6	
05/04/2023		1.0	
05/05/2023		1.0	
05/08/2023		1.0	
05/09/2023		2.0	
05/10/2023		1.0	
05/11/2023		1.0	
05/12/2023		1.0	
05/13/2023		1.0	
05/15/2023	Elective cesarean + general anesthesia		No major complications
Postpartum (D1)	Maintained eltrombopag + IVIG	3.0	No bleeding, good recovery
05/17/2023		18.0	
05/18/2023		4.0	
05/19/2023		7.0	
05/20/2023		2.0	
05/21/2023	Hospital discharge		Stabilized; no outpatient follow-up

**Table 2:** Newborn case data.

Date/day of life	Management/event	Platelets (× 10^3^/mm^3^)	Observations
Birth (D0)	Male newborn, 2450 g, Apgar 8/9/10	32	Cervical hematoma
D1	Platelet transfusion (15 mL/kg) + IVIG 0.5 g/kg		Good clinical evolution
D4	IVIG 2 g/kg in divided doses	37	No further transfusion needed
∼D7	Hospital discharge	60	Outpatient follow-up recommended

## Data Availability

Data sharing is not applicable to this article as no new data were created or analyzed in this study.
